# Plasma thrombin-activatable fibrinolysis inhibitor (TAFI) antigen levels in acromegaly patients in remission

**DOI:** 10.3906/sag-1812-231

**Published:** 2019-10-24

**Authors:** Mehmet ERDOĞAN, Mustafa ÖZBEK, Erdem AKBAL, Kemal ÜRETEN

**Affiliations:** 1 Department of Endocrinology and Metabolism Diseases, Faculty of Medicine, Ege University, İzmir Turkey; 2 Endocrinology and Metabolism Diseases Ministry of Health Dışkapı Yıldırım Beyazıt Education and Research Hospital, Ankara Turkey; 3 Department of Internal Medicine, Faculty of Medicine, Çanakkale Onsekiz Mart University, Çanakkale Turkey; 4 Department of Rheumatology, Faculty of Medicine, Kırıkkale University, Kırıkkale Turkey

**Keywords:** TAFI antigen, acromegaly, cardiovascular disease

## Abstract

**Background/aim:**

Acromegaly is associated with increased morbidity and mortality, mostly due to cardiovascular complications. Plasma thrombin-activatable fibrinolysis inhibitor (TAFI) antigen levels are associated with coagulation/fibrinolysis and inflammation. Plasma TAFI may play a role in arterial thrombosis in cardiovascular diseases. In this study, it was aimed to evaluate the thrombin-activatable fibrinolysis inhibitor (TAFI) antigen and homocysteine levels in patients with acromegaly and healthy control subjects.

**Materials and methods:**

Plasma TAFI antigen and homocysteine levels in 29 consecutive patients with acromegaly and 26 age-matched healthy control subjects were measured. All patients included in the study were in remission. The TAFIa/ai antigen in the plasma samples was measured using a commercially available ELISA kit.

**Results:**

Routine biochemical parameters, fasting blood glucose, prolactin, thyroid stimulating hormone, total-cholesterol, low density lipoprotein cholesterol, triglyceride, and homocysteine levels were similar in the 2 groups (P > 0.05), whereas the plasma TAFI antigen levels were significantly elevated in the acromegalic patients (154.7 ± 94.0%) when compared with the control subjects (107.2 ± 61.6%) (P = 0.033). No significant correlation was identified by Pearson’s correlation test between the plasma TAFI antigen and homocysteine levels (r = 0.320, P = 0.250).

**Conclusion:**

A significant alteration in the plasma TAFI antigen levels was detected in acromegaly. Increased plasma TAFI antigen levels might aggravate prothrombotic and thrombotic events in patients with acromegaly.

## 1. Introduction 

Acromegaly is a disease caused by the overproduction of growth hormone (GH) from a GH-secreting pituitary adenoma. GH and insulin-like growth factor-I (IGF-I) excess are associated with increased morbidity and mortality [1,2]. Exposure to high levels of GH is associated with an increased cardiovascular risk profile due to cardiac abnormalities, insulin resistance, dyslipidemia, and obesity [3–5]. 

A new plasma protein, that may play an important regulatory role in fibrinolysis, has been identified as a carboxypeptidase B-like proenzyme [6,7] that can be activated by thrombin. Upon activation by thrombin or plasma, it is converted into an active enzyme thrombin-activatable fibrinolysis inhibitor (TAFI), which acts as an inhibitor of tissue-type plasminogen activator dependent fibrinolysis. It decreases plasmin formation by removing lysine residues from the fibrin surface [8]. The potential physiological role of TAFI in the fibrinolytic process has been underscored by numerous in vitro and in vivo data [9,10]. It also seems to be a potential hemostatic risk factor for coronary artery disease (CAD). TAFI is associated with coagulation/fibrinolysis and inflammation, plasma TAFI may also participate in arterial thrombosis in cardiovascular diseases (CVDs) [11]. Increased plasma TAFI levels were observed in obese and type 2 diabetic patients, and those with insulin resistance [12,13].

Homocysteine and its cyclic ester homocysteine thiolactone (HTL) have been involved in the detrimental consequences associated with hyperhomocysteinemia, an independent risk factor for vascular diseases [14]. Moreover, a rise in homocysteine has been associated with the risk of CVD, in both remission and active acromegaly disease groups after surgery [15]. Increased homocysteine and TAFI comprise similar clinical importance in terms of CVD.

There is not enough information regarding the status of the fibrinolytic system in acromegaly. Plasma TAFI activity may be related to endogenous coagulation/fibrinolysis in patients with acromegaly. Herein, it was aimed to evaluate the relation between the TAFI antigen and homocysteine levels in acromegaly. 

## 2. Materials and methods 

Twenty nine patients with acromegaly (20 females, 9 males; average age of 44.7 ± 10.2 years, average body mass index (BMI) of 24.4 ± 5.4 kg/m², average duration of disease was 5.9 ± 0.7 years). All patients included in the study were in remission.

The controls included 26 age-matched healthy subjects (20 females and 6 males; aged 43.4 ± 9.6 years, BMI of 23.4 ± 5.01 kg/m²). The control group consisted of healthy hospital staff. None of the participants had a medical history of chronic disease, such as morbid obesity, hypertension, familial hyperlipidemia, diabetes mellitus, chronic renal failure, chronic hepatic disease, CVDs, or coagulation disorders. 

 Patients were excluded if they had been diagnosed with hepatic or renal dysfunction, or if they had a history of severe systemic disease or malignancy. Patients taking lipid lowering drugs, angiotensin-2 receptor blockers, angiotensin converting enzyme inhibitors, oral contraceptives, estrogen, or any other drugs that may have influenced coagulation or the fibrinolytic system (such as anticoagulants and antiplatelet agents) were also not included in the study. Patients with diabetes mellitus, cardiac abnormalities, hypertension, dyslipidemia, or hypopituitarism, or active or uncontrolled acromegaly patients were excluded from the study. Smokers were also not included in the study.

The diagnosis of acromegaly was based on the Endocrine Society Clinical Practice Guidelines [16], as follows: 1) typical acromegaly clinical manifestations; 2) serum nadir GH concentration of >1 ng/mL after a 75-g oral glucose tolerance test (OGTT) or fasting GH value of >2.5 ng/mL; 3) serum IGF-1 levels above the normal age-adjusted range; and 4) enhanced magnetic resonance imaging showing a pituitary tumor in the sellar area. In addition, every patient underwent surgery, and the presence of a pituitary adenoma was confirmed by a postoperative pathological examination.

All acromegaly patients underwent microsurgical selective resection. Remission was achieved in 21 of the 29 patients (72.4%) after surgery alone. Due to elevated GH and IGF-I levels after microsurgery (radiotherapy and/or medical therapy, octreotide-LAR and/or lanreotide-LAR and cabergoline), 8 patients (27.5%) were receiving combination therapy. The patients did not take on any medication for acromegaly during the study. Acromegaly was considered in remission when the glucose suppressed GH levels were below 0.4 ng/mL and IGF-1 levels were normal for their age and sex [17].

This study was approved by the Ethical Committee and all participants provided their informed consent before participation. The study was carried out in the Department of Endocrinology and Metabolism Diseases at the Dışkapı Education and Research Hospital in Ankara. Sufficient volume of the plasma was stored at –30 °C to establish concentrations of TAFI antigen. The TAFIa/ai antigen in the plasma samples was measured with a commercially available enzyme-linked immunosorbent assay kit (Imuclone TAFI, American Diagnostica Inc. Stamford, CT, USA). The intra- and interassay coefficients of variation were 6% and 10%, respectively. Basal serum GH levels were determined by chemiluminescent immunometric assay (ICMA, Immulite 2000, DPC, Siemens Diagnostics, Los Angeles, USA). Serum IGF-I levels were determined by radioimmunoassay. Serum homocysteine levels were measured using Immulite 2000 (Siemens, medical solutions diagnostics, USA). A fasting venous blood sample was obtained for the glucose (hexokinase method using the Autoanalyser AU5200), triglyceride (glycerophosphate oxidase method using the Autoanalyser AU5200), total cholesterol (TC), low-density lipoprotein (LDL), and high-density lipoprotein (HDL) cholesterol (spectrophotometric method using Roche-Hitachi Modular P device) measurements. 

### 2.1. Statistical analyses 

The significance of the differences in the averages between the groups was evaluated using Student’s t test and the Mann–Whitney U test. The descriptive statistics were shown as the mean ± standard deviation for the continuous variables. Normal distribution of the numerical variability was examined using the Shapiro–Wilk test. Correlations between the parameters were tested using Pearson’s correlation analysis. The presence of a directional relation between the plasma TAFI antigen and homocysteine levels was evaluated using a correlation test. SPSS v.25 (IBM Corp, Armonk, NY, USA) was used for the statistical analysis. P < 0.05 was considered statistically significant.

## 3. Results 

The biochemical parameters and clinical characteristics of the patients with acromegaly and the control subjects are shown in the Table. Routine biochemical parameters including fasting blood glucose, prolactin, thyroid stimulating hormone (TSH), TC, LDL-cholesterol (LDL-C), triglycerides (TG), homocysteine, and GH/IGF-I were similar in the 2 groups (P >0.05), whereas the plasma TAFI antigen levels were significantly elevated in the patients with acromegaly (154.7 ± 94.0%) when compared with the control subjects (107.2 ± 61.6%) (P = 0.033). In patients with acromegaly, the average basal plasma GH and IGF-I levels were 1.40 ± 1.10 ng/mL (normal reference range, 0.05–10.0 ng/mL) and 253.0 ± 219.10 ng/mL (normal reference range, 169–591 ng/mL), respectively. No significant correlation was identified by Pearson’s correlation test between the plasma TAFI antigen and homocysteine levels (r = 0.320, P = 0.250).

## 4. Discussion 

In patients with acromegaly, the mortality rate is two- to four-fold increase, related predominantly to CVD [18]. High serum GH levels seem to be more consistently independent predictors of mortality than IGF-I levels [19]. The plasma homocysteine and TAFI antigen levels were measured in the patients with acromegaly and the healthy subjects. There was no significant difference between the plasma homocysteine levels in patients with acromegaly (9.60 ± 2.80 mmol/L) and the control subjects (10.20 ± 4.30 mmol/L). Likewise, Kirilov et al. reported that the total homocysteine levels, as biochemical markers of endothelial dysfunction and atherosclerosis in patients with active and cured acromegaly, did not differ significantly in the studied groups [20]. However, Hekimsoy et al. demonstrated that acromegaly patients with high GH levels after an OGTT had much higher levels of homocysteine than patients with lower GH levels [21]. These findings demonstrated that further studies are needed to consider hyperhomocysteinemia as an independent risk factor for CVD in acromegaly. The plasma TAFI antigen levels of patients with CAD, measured in different studies, were mostly increased [22,23], but also decreased [24] as well. TAFI antigen levels were approximately 4 times higher in patients with CAD than in healthy controls, in a previous study [25]. TAFI antigen levels were also found to be significantly higher in males with CAD when compared to healthy controls [26]. Similarly, plasma TAFI levels were higher in patients with type 2 diabetes than in healthy controls, and plasma TAFI levels were shown to be associated with other diseases exhibiting insulin resistance. Solomon et al. reported higher plasma TAFI antigen levels in obese type 2 diabetes patients than in nonobese type 2 diabetics [27]. Oral et al. reported that homeostatic parameters and plasma TAFI antigen levels were higher in patients with polycystic ovary syndrome when compared with the control subjects [28]. Factor VII, TAFI antigen, and thrombomodulin levels were higher in patients with hypothyroidism than in healthy subjects in another study, causally suggesting increased cardiovascular mortality in hypothyroidism [29]. However, there is no adequate information about plasma TAFI antigen levels in acromegaly disease, unlike other hemostatic and thrombotic parameters. Erem et al. reported that plasma TAFI antigen levels did not significantly change in patients with active acromegaly when compared with the controls [30]. The plasma TAFI antigen levels in the current study were statistically higher in patients with acromegaly in remission (154.7 ± 94.0%) than in the healthy control subjects (107.2 ± 61.6%) (P = 0.033). Our study confirmed that increased plasma TAFI antigen levels were detected in patients with acromegaly. One limitation of this study was that a group of active acromegaly patients was not included in the study. The other limitation was only including nonsmokers as participants. 

In conclusion, the present study was a case control study in which significant alterations in the plasma TAFI antigen levels were detected in patients with acromegaly in remission. Plasma TAFI antigen levels might be associated with the prothrombotic state in patients with acromegaly. Further studies with more similar groups are needed to confirm the results.
